# Ebola virus disease surveillance and response preparedness in northern Ghana

**DOI:** 10.3402/gha.v9.29763

**Published:** 2016-05-03

**Authors:** Martin N. Adokiya, John K. Awoonor-Williams

**Affiliations:** 1 Department of Community Health, School of Allied Health Sciences, University for Development Studies, Tamale, Ghana; 2 Regional Health Directorate, Ghana Health Service, Upper East Region, Bolgatanga, Ghana; 3 Swiss Tropical and Public Health Institute, University of Basel, Basel, Switzerland

**Keywords:** Ebola, surveillance, core and support functions, health workers Ghana

## Abstract

**Background:**

The recent Ebola virus disease (EVD) outbreak has been described as unprecedented in terms of morbidity, mortality, and geographical extension. It also revealed many weaknesses and inadequacies for disease surveillance and response systems in Africa due to underqualified staff, cultural beliefs, and lack of trust for the formal health care sector. In 2014, Ghana had high risk of importation of EVD cases.

**Objective:**

The objective of this study was to assess the EVD surveillance and response system in northern Ghana.

**Design:**

This was an observational study conducted among 47 health workers (district directors, medical, disease control, and laboratory officers) in all 13 districts of the Upper East Region representing public, mission, and private health services. A semi-structured questionnaire with focus on core and support functions (e.g. detection, confirmation) was administered to the informants. Their responses were recorded according to specific themes. In addition, 34 weekly Integrated Disease Surveillance and Response reports (August 2014 to March 2015) were collated from each district.

**Results:**

In 2014 and 2015, a total of 10 suspected Ebola cases were clinically diagnosed from four districts. Out of the suspected cases, eight died and the cause of death was unexplained. All the 10 suspected cases were reported, none was confirmed. The informants had knowledge on EVD surveillance and data reporting. However, there were gaps such as delayed reporting, low quality protective equipment (e.g. gloves, aprons), inadequate staff, and lack of laboratory capacity. The majority (38/47) of the respondents were not satisfied with EVD surveillance system and response preparedness due to lack of infrared thermometers, ineffective screening, and lack of isolation centres.

**Conclusion:**

EVD surveillance and response preparedness is insufficient and the epidemic is a wake-up call for early detection and response preparedness. Ebola surveillance remains a neglected public health issue. Thus, disease surveillance strengthening is urgently needed in Ghana.

## Introduction

In December 2013, the largest Ebola virus disease (EVD) outbreak began in West Africa (1). The current Ebola outbreak has been described as unprecedented and its public health impact in terms of morbidity, mortality, and geographical extension has been far greater than previously experienced (1–3). Since September 2014, Ghana had the highest probability of importation of EVD cases via travellers from the most affected countries (traveller volume of 25,272 by air travel) and followed closely by Senegal (traveller volume of 20,818) in Africa (3). This outbreak has revealed many weaknesses and inadequacies for disease surveillance and response systems in developing countries. Low-income countries often lack the needed health infrastructure to prevent and control an outbreak (4). For instance, the most affected countries in West Africa lack adequate laboratory, public health, clinical personnel, appropriate equipment, supplies, and protocol as well as qualified health workers (4). The effects of the outbreak on the already weakened public services and health systems has resulted in further crippling of the health sector and increased the impact of other endemic diseases and their associated deaths (1, 5).

Disease surveillance is the ongoing systematic collection, analysis, and interpretation of health data essential for planning, implementation, and evaluation of public health practice, closely integrated with timely dissemination of these data to those who need to know (6, 7). In spite of increased efforts in strengthening health systems, many countries still do not possess the requisite core health system capacities (4). In particular, the purpose of International Health Regulations (IHR 2005) is to prevent, protect against, control, and provide public health response to the international spread of disease in ways that are vital and limited to public health risk (8, 9). As a result, planning and decision-making is based on unreliable and low quality data (10, 11). In view of this, the World Health Organization Regional Office for Africa (WHO-AFRO) and other partners were asked by the various ministries of health to develop strategies that would enable countries to prepare and respond adequately and overall strengthen surveillance capacities (12). In 1998, WHO-AFRO adopted the Integrated Disease Surveillance and Response (IDSR) strategy for its member countries as a comprehensive public health strategy (8, 13–17). The goal of the IDSR strategy is to build member countries capacity to detect, report, and effectively respond to priority diseases (e.g. Ebola) as well as to link laboratory activities for public health action (14, 18–20). In Ghana, Ebola is one of the 43 diseases that are reported under the surveillance system (9, 20).

### EVD response preparedness in Ghana

In Ghana, the Public Health Emergency Management Committees (PHEMC) at the regional and district levels are responsible for overseeing and implementing the epidemic response plans. The PHEMCs are coordination committees composed of technical and non-technical members (e.g. municipal/district chief executive, police commander, director of health service, public health nurse, disease control officer, environmental health officer, medical or clinical officer, veterinary experts, laboratory technician/technologist, and Red Cross) (8). A Rapid Response Team (RRT) is a technical, multi-disciplinary team that is readily available for mobilisation and deployment in cases of emergencies. The members of the epidemic RRT include a disease control officer, laboratory technician, clinician, environmental health officer, veterinary, and others, based on availability of the technical staff and specificity of the outbreak. For EVD response preparedness, the district RRT is responsible for public sensitisation, clinician sensitisation, and active surveillance. It includes investigation of rumours, outbreaks, and other public health emergencies; proposing control measures including risk communication activities; and coordinating rapid response (8).

In Ghana, the EVD response plan had the following components: risk communication-social mobilisation and health education, epidemiological and laboratory surveillance, case management, logistics, security, and financial resources and coordination. In implementing the plan, public sensitisation, clinician sensitisation, and active surveillance are carried out with key messages (e.g. the immediate seeking of health care when EVD is suspected). Frontline health workers are trained to identify any suspected cases. The response teams are on the alert to pick, package, and transport specimens to the reference laboratory for confirmation. The core members of the response team include the municipal/district director of health service and disease control, laboratory, and health promotion officers. The laboratory officer is responsible for collecting specimens, document appropriately, store, perform laboratory analysis for presumptive diagnosis, provide results to clinical staff and patients, report results to disease control office, and report observed changes in trends during routine analysis of specimens in the laboratory. The medical officer/clinician is responsible for ensuring the availability of isolation wards for suspected EVD cases, keeping suspected cases at the hospital or health facility for the rapid response team, and reporting suspected cases to the district director for planning and decision-making as well as the provision of needed logistics. The objective of this study was to assess the EVD surveillance and response system in northern Ghana.

## Methods

### Study setting

Ghana is situated in West Africa and bordered by Ivory Coast to the west, Burkina Faso to the north, Togo to the east, and the Atlantic Ocean to the south. It has a total land area of 238,537 km^2^. The country is composed of 10 administrative regions and 216 districts and a population of about 25 million in 2010 (11, 21, 22). The Upper East Region (UER) is bordered by Burkina Faso to the north and Togo to the east (11, 23, 24). It is made of three Municipals (Bolgatanga, Kassena-Nankana, and Bawku) and 10 districts (Builsa North, Builsa South, Kassena-Nankana West, Bongo, Garu-Tempane, Talensi, Nabdam, Bawku West, Binduri, and Pusiga). The ecological characteristic of the Region is that of Sahel with semi-arid Guinea savannah vegetation. There is one rainy season in each year from June to October. The economy of Ghana has undergone minimal changes over the past two decades (22). According to the World Bank, the annual Gross Domestic Product (GDP) growth rate of Ghana was 4.8% in 2009 and increased until it reached 14.0% in 2011. From 2012, with an annual GDP growth rate of 9.3%, the economy declined further to 3.4% in 2015 (25). Between 2013 and 2014, the GDP declined from USD48.6 billion dollars in 2013 (7.3% annual growth rate) to USD38.6 billion in 2014 (4.2% annual growth rate) (22, 25). The annual GDP growth rate of 4.2% in 2014 was even lower than the 5.9% reported in 2005. At the national level, the agriculture sector has been overtaken by the service and industry sectors. By 2014, the service sector (52%) and industry sector (27%) contributed nearly 80% of the GDP (22). The common export commodities include cocoa, gold, and timber. In recent years, the economy has slightly diversified to include export of non-traditional commodities such as pineapples, bananas, yams, and cashew nuts (22). In 2013, the total health expenditure was nearly 10% of the GDP in 2013. However in the UER, the local economy depends on subsistence agriculture and the crops commonly cultivated include millet, maize, sorghum, and rice (20, 26). The majority of the people live in rural settings, and households are grouped into extended family units/compounds (27). In Ghana, the infant mortality rate is 41 deaths per 1,000 live births and the under 5-years of age mortality is slightly above 60 deaths per 1,000 live births. In UER, infant mortality rate is 46 deaths per 1,000 live births and the under-5 years of age mortality rate is 72 deaths per 1,000 live births, which are above the national rates (22). Ghana has one of the lowest total fertility rates (4.2 children per woman) in sub-Saharan Africa (SSA). It decline from 6.4 children per woman in 1988 to 4.2 children per woman in 2014 (22).

### Ghana health care system

Ghana's health care system is organised in a three-tier system (i.e. district, regional, and national). This is further structured into five levels; community, sub-district, district, regional, and national (20). The smallest unit of the health system is the community-based health planning and services (CHPS), which is responsible for the provision of community level health activities including the treatment of minor ailments, home-visits, community outreaches, education and health promotion (20, 28). The Primary Health Care (PHC) system is delivered by the district health system and entails all institutions including CHPS, health centres, clinics, and hospitals (20). In Ghana, each district is served or supposed to be served by a hospital, several health centres, private and mission clinics, and CHPS Compounds (20, 28). The district hospital serves as the first referral point in the PHC system in the country (20, 28, 29), where clinical, surgical, laboratory, and maternity services are provided. For the purpose of surveillance, all institutions (public, private, non-governmental organisations, etc.) with outpatient and/or inpatient facilities are described as health facilities. A sub-district is a health implementation unit within the district that generally serves a maximum population of 30,000 people. A district is described as a decentralised administrative unit in the country that serves a population of 80,000 to 120,000 people (8). The region is the main administrative unit with a number of decentralised districts (e.g. 13 districts in UER), while the national level of administration is where policies are set and resources are allocated. In relation to surveillance, the national level reports on priority diseases based on a decision instrument to report events of public health concern to WHO. Malaria, gastroenteritis, and acute respiratory infection are the main causes of morbidity and mortality in the study area, and outbreaks of meningitis also occur periodically (20, 26).

### Surveillance and diagnostic procedures in Ghana

At the health facility level, standard case definitions are used to identify suspected cases. According to the Ghana IDSR technical guidelines (May 2011), Ebola or Marburg viral haemorrhagic fevers are classified as suspected or confirmed cases. ‘A suspected case is any person reporting with fever and has no response to usual treatment in the area, and at least one of the following signs: bloody diarrhoea, bleed from gums, bleeding into skin (purpura), bleeding into eyes and urine’ (9). ‘A confirmed case is any suspected case with laboratory confirmation (positive IgM antibody, positive Polymerase Chain Reaction or viral isolation), or epidemiologic link to confirmed cases or outbreak’ (9). These definitions are general and lack details and specifics. A suspected case of Ebola or Marburg viral haemorrhagic fever is confirmed by laboratory confirmation using positive IgM antibody, positive polymerase chain reaction (PCR) or viral isolation, or epidemiologic link to confirmed cases or outbreak (9). The information includes suspected, laboratory confirmed cases, and deaths (20, 29, 30).

The standard case definitions in the IDSR technical guidelines are a set of criteria used to decide whether a person has a particular disease or condition (9, 20). However, there are several diseases with similar signs and symptoms. Thus, biological specimens are required to be collected, stored, and processed to achieve specific diagnoses (e.g. malaria and suspected Ebola) (9). For suspected diseases for which a health centre/clinic lacks the capacity to perform laboratory tests for confirmation, specimens are sent to the district hospital or the district health administration (DHA) for onward delivery at a designated reference laboratory (e.g. meningitis or Ebola) (20). The specimens are transported from health facilities or district or regional levels to the reference laboratories. In Ghana, the important referral laboratories include the Noguchi Memorial Institute for Medical Research (NMIMR) and the National Public Health Reference Laboratories in Accra (e.g. polio and Ebola).

### Ebola case definition during the outbreak in West Africa

During the peak of Ebola outbreak in West Africa, the health system in Ghana revised its Ebola case definition to reflect the prevailing situation. This information was then distributed to all levels of the health system across the country.

*Alert case*: any ill person with onset of fever and not responding to treatment for common causes of fever in the area and or any ill person with at least one of the following signs: bleeding, bloody diarrhoea, blood in urine, or any sudden death.

*Suspected case*: any person alive or dead, suffering or having suffered from a sudden onset of high fever and having had contact with a suspected, probable or confirmed Ebola case; or any person with sudden onset of high fever and at least three of the following symptoms: headache, anorexia (loss of appetite), lethargy, aching muscles or joints, breathing difficulties, vomiting, diarrhoea, stomach, difficulty swallowing, and hiccups. A suspected case is also any person with inexplicable bleeding or any sudden inexplicable death.

*Probable case*: any deceased suspected case (if it has not been possible to collect specimens for laboratory confirmation) having an epidemiological link with a confirmed Ebola case or any suspected case evaluated by a clinician.

*Confirmed case*: any suspected or probable case with a positive laboratory result; laboratory confirmed cases must test positive for the virus antigen, either by detection of virus ribonucleic acid (RNA) by reverse transcriptase-PCR (RT-PCR), or by detection of IgM antibodies directed against Ebola.

### Study design

This was an observational cross-sectional study conducted among 47 frontline health workers in all the 13 districts of UER to query key informants who are responsible for health care delivery at the district level. The definition of frontline health worker in this study includes permanent staff such as medical doctors, directors of district health services, disease control/surveillance officers, and laboratory officers) of public, private, and mission hospitals and the DHA. Interviews were conducted on the core and support functions of disease surveillance and satisfaction of the Ebola surveillance system using a semi-structured questionnaire. The UER is one of the three northern regions of Ghana. Bolgatanga is the regional capital of UER and it is about 782 km from the Accra (national capital of Ghana). The distance from Accra to Paga border (the Ghana–Burkina Faso border) is about 822 km while Accra to Pusiga border (Ghana–Togo border) is about 860 km. There is one main standard road from Accra through Bolgatanga and Paga to Burkina Faso, while the road leading from Bolgatanga to Togo is in a poor state and partly untarred. Often, it takes about 12–15 hours to travel by road from Accra to either border. Thus, the region is remote from the national capital and has a higher likelihood of Ebola importation due to its borders.

### Key informants selection

At each district, one medical officer or medical/physician assistant, one laboratory officer, one disease control officer, and one director of DHA participated in the study. Ideally, each DHA is supposed to have district officers (e.g. director of health service, medical officer, disease control officer and laboratory officer). However, some districts do not have district hospitals and medical officers such as Builsa South, Binduri, and Nabdam. In addition, the Binduri and Nabdam districts do not have laboratory officers. In each district, the above officers are standard required staff for the health system. The responsibilities during the peak of Ebola outbreak remained the same. The medical officer is responsible for generating surveillance information using clinical diagnosis. When a person visits any health facility such as a hospital, health centre, clinic, or CHPS Compound to seek health care, the personal information of the person is collected and recorded in a register at the Out-Patient Department. Then the person goes to the consulting room to be diagnosed by a medical officer or physician assistant. In the consulting room, additional information is collected from the person. This information is recorded in the consulting room register as provisional diagnosis. The suspected case is then referred to the laboratory for further investigation and confirmation depending on the signs and symptoms (20). There are standard criteria for referring patients to the laboratory. For example, all suspected malaria cases must be referred to the laboratory for testing. The laboratory officer is responsible for taking the samples for investigation and further analysis. After the samples from suspected cases have been tested in the laboratory, the medical officer updates the consulting room register with the information of principal diagnosis and/or additional diagnosis. The disease control officer collates daily, the number of suspected cases, confirmed cases and deaths using the consulting room registers, laboratory registers and in-patient registers as sources of data. This information is submitted to the DHA using the weekly and monthly IDSR reporting forms. They also conduct case contact tracing in the communities. The health administration director is responsible for planning and decision-making at the district health system.

Eligibility for the key informants included: 1) work with the district health system; 2) familiarity and active involvement or likelihood of involvement when EVD is reported; 3) willingness to participate; and 4) completion of written consent were required before the interviews were conducted. On the day of the field visit, they were interviewed using a semi-structured questionnaire. If any of them were not available on the day of the visit, arrangements were made for a revisit. However, new employees (less than 3 months in a position) were excluded. The older employees were included in order that they may have adequate and accurate information regarding changes on disease surveillance before and during the Ebola outbreak in West Africa.

### Data collection

A total of 47 key informant interviews were conducted. The main issues addressed in the questionnaire were 1) core functions of the surveillance system (e.g. detection and registration, and response); 2) support functions (e.g. training) and 3) satisfaction and challenges with Ebola surveillance. Examples of the questions included ‘What are the signs and symptom for suspected EVD? How did your district or region investigate rumours of suspected Ebola cases?’ In addition, 34 weekly IDSR reports (August 2014 to March 2015) were collated from each district. The research team considered August 2014 as the peak of the outbreak with high risk of spread to other countries including Ghana. Two assistants were trained and they assisted with the data collection from 25 May to 6 June 2015.

### Data analysis

Only frequencies of suspected cases were produced from the IDSR weekly reports. For the qualitative data analysis, the responses of the informants were transcribed from the semi-structured questionnaire, and the transcripts were read multiple of times to identify critical responses on the major components of the EVD responses preparedness as well as specific themes based on the IDSR technical guidelines, such as surveillance core functions (detection, registration, confirmation, reporting, analysis, and epidemic preparedness) and support functions (supervision, training, and resources). The perspective of the key informants on the state of the EVD surveillance preparedness and response were also captured.

### Ethical considerations

Individual written informed consent was obtained before the interviews were conducted. The Navrongo Health Research Centre Institutional Review Board (NHRCIRB155) granted ethical approval. Permission was also received from the regional health directorate of Ghana Health Service.

## Results

### Background information on respondents

Table 1 shows the background information of the informants. The majority of the informants interviewed were males (39/47). All the females were district directors of health service. Among the category of directors, females were the majority (8/13). The remaining 34 informants were males working as medical officers (10), disease control officers (13), laboratory officers (10), and a hospital administrator (1).

**Table 1:** Background information of respondents in Upper East Region Ghana

	Position of respondent	Gender	Frequency
1.	Municipal Directors of Ghana Health Service	Males Female	1 2
2.	District Directors of Ghana Health Service	Male Female	4 6
3.	Municipal Disease Control Officers	Male Female	3 0
4.	District Disease Control Officers	Male Female	10 0
5.	Medical Officers of Municipal Hospital	Male Female	3 0
3.	Medical Officers of District Hospital	Male Female	7 0
4	Staff of Hospital Laboratory Officers	Male Female	10 0
5.	Hospital General Manager	Male	1

### Quantitative data

Table 2 shows weekly IDSR data over the 34 weeks of clinically diagnosed EVD cases. Four out of the 13 districts reported 10 EVD cases. The Bawku West district and Bawku Municipal accounted for the majority of the cases and eight of the suspected cases died. All the suspected EVD cases were identified due to their bleeding state (e.g. snake bite, bleeding due to pregnancy, and coughing with blood due to tuberculosis) or having high temperature above 38°C. The causes of death among the suspected cases were unexplained. Bawku Municipal was the only district that reported a case in 2015. The 10 suspected cases that were reported, none was confirmed (i.e. positive for the virus antigen).

**Table 2:** Reported cases of suspected EVD in Upper East Region (2014 and 2015), Ghana

Acute haemorrhagic fever (e.g. Ebola)		2014 (week number 32–52)			2015 (week number 01–13)		
		
Districts	Population in 2014	Suspected	Confirmed	Death	Suspected	Confirmed	Death
Bawku Municipal	103,354	3	0	3	1	0	0
Bawku West	98,630	4	0	3	0	0	0
Binduri	64,585	0	0	0	0	0	0
Bolgatanga Municipal	137,979	1	0	1	0	0	0
Bongo	88,677	0	0	0	0	0	0
Builsa North	59,237	0	0	0	0	0	0
Builsa South	38,298	0	0	0	0	0	0
Garu Tempane	136,356	0	0	0	0	0	0
Kassena Nankana Municipal	115,317	1	0	1	0	0	0
Kassena Nankana West	74,121	0	0	0	0	0	0
Nabdam	35,479	0	0	0	0	0	0
Pusiga	60,496	0	0	0	0	0	0
Talensi	85,162	0	0	0	0	0	0
Upper East Region	1,097,691	9	0	8	1	0	0

### Qualitative data

In total, 47 key informants were interviewed on EVD surveillance and response preparedness. The informants included 10 medical officers/physician assistants, 13 district directors, 13 disease control officers, 10 laboratory officers and one hospital administrator. Generally, their answers were similar, and the EVD surveillance challenges were cross-cutting in the region.

### Overall EVD surveillance and response preparedness

The informants had mixed views on the Ebola surveillance. Less than half (6 of 13) of districts had public hospitals which is below the required number. The remaining districts are served by health centres and private hospitals. Only eight of the workers said that they were satisfied with EVD surveillance system. The majority were not satisfied with the surveillance system due to lack of infra-red thermometers, inadequate equipment, ineffective screening, inadequate staff, and lack of isolation centres.

We did not even have infra-red thermometers at the peak of the epidemic and we still have gaps in terms of PPEs; the waiting centre at the border is a small room with no fence and there are only two beds in that isolation centre. (District Director of Health Service 3)

The surveillance system is okay but the screening for suspected EVD cases is ineffective. There are no gloves for workers. If there was one EVD case, all the PPEs will be used and they were even of low quality. If Ebola happens in Ghana, we will not survive it. (Municipal Disease Control Officer 1)

### EVD detection and confirmation

The majority (46/47) of the informants had general knowledge on Ebola surveillance. However, less than half (20/47) described the existing surveillance system to be functional. They reported that the thermometers were inaccurate resulting in the under- or over-estimation of suspected cases since high temperature is the main key detection criterion. There were no specific registers for recording of EVD data in the districts.

It was difficult to differentiate Ebola suspected cases from malaria. The emphasis was on the high temperature and it was too sensitive to be used for Ebola. It meant that everyone who came to the hospital was suspected of Ebola. In addition, the thermometer calibration had problems. Some of the thermometers were under reading the temperature of patients. (District Medical Officer 5)

Over half (33/47) of the informants reported that there were problems with screening and recording of suspected EVD cases. These included absolute no screening, inadequate logistics, inadequate health workers, and irregular use of personal protective equipment (PPE). All the districts had no capacity to confirm suspected EVD cases using laboratory procedures. Besides, there were other challenges such as fear among health workers, lack of holding centres and cultural barriers (e.g. immediate burials, touching of the dead by relatives).

The PPEs are not regularly used and they are of low quality. We heard of fear among health workers who were supposed to take the specimens from suspected cases in Bolgatanga. In addition, there is no isolation place for suspected cases. Patients come to the OPD (Out Patient Department) and sit together which exposes everyone to the disease if there were true Ebola cases. (District Director of Health Service 1)

### EVD data reporting, feedback, and response preparedness

Nearly three-quarters (34/47) of the informants reported that, the routine disease surveillance data reporting was good and that they regularly submitted weekly and monthly IDSR reports. Paper-based surveillance data, is sent from the health facilities to the DHA, but for suspected Ebola cases, the information was largely transmitted by mobile phone. Challenges of EVD data reporting included non-involvement of laboratory staff, lack of transport medium for specimens, delays, irregular reporting and inadequate staff.

The Ebola reporting is not different from other diseases except that more attention was given to it. For example, there was rapid response from the region when suspected cases were reported compared to other diseases like tuberculosis. (District Disease Control Officer 3)

Problems of reporting are inadequate means of transport and lack of transport medium for specimens from the district to the region. The district has human resource and internet service challenges which hamper surveillance data reporting. (District Laboratory Officer 1)

About half (25/47) of the interviewees indicated that, general surveillance data was analysed at the district level but often not posted on the notice board for the public. The majority (41/47) of the informants indicated that there is a response system in place for suspected EVD cases. The response include specimen taking and reporting to the relevant authorities for appropriate public health action. About three-quarters (34/47) of informants reported that there is a feedback on the suspected EVD cases in the form of bulletin through email system to the districts from the region. However, written reports were irregular. Apart from investigations by phone in case of rumours, feedback, and information on EVD surveillance to health facilities only took place during the monthly unit head meetings or bi-annual or annual review meetings.

We had three suspected cases in the district. When we got the rumors, we followed to the communities to identify the index case, the close contacts and to identify if others had developed similar signs and symptoms. (District Director of Health Service 5)

Generally, feedback on disease surveillance to health facilities is often delayed until monthly, quarterly or bi-annual review meetings. (District Medical Officer 10)

### EVD surveillance supervision and training

The majority of the respondents (36/47) indicated that the districts or hospitals have been supervised by the regional health directorate or national level. However, these visits were irregular and also not purposely for EVD surveillance. All the districts had a trained disease control officers. Only a minority (7/47) of the informants were not trained on Ebola. The challenges associated with training were inadequate knowledge, inadequate funding and lack of interest.

Immediately the first suspected EVD case was reported, there was a team from the national level to the region. It was a single visit on HIV/AIDS but they added Ebola during the discussion. (Municipal Medical Officer 3)

We had training at the region on general IDSR including Ebola which was organized by regional health administration. The training was on what happened in Liberia, Sierra Leone and Guinea based on the Ghanaian delegation that visited those countries to learn and support them to control the outbreak. (District Disease Control Officer 4)

### EVD surveillance resources and satisfaction

Nearly all the informants (45/47) reported that the districts received some resources (e.g. posters, gloves, infra-red thermometers, aprons, bleaches, and sanitizers). According to the informants, the PPEs were of low quality and inadequate. Generally, the majority (38/47) of the informants said that the Ebola surveillance system was not well-functioning and unsatisfactory. The reasons included inadequate resources and ineffective screening.

We received PPEs such as overalls, aprons, gloves and sanitizers. The overalls that were given are low quality compared to what was used in the three most affected countries according to the regional team that visited those countries. (District Director of Health Service 3)

We are a country with strategies and plans, but we execute none of them. The plans regarding Ebola prevention and quality of PPEs that were provided shows clearly that we were not ready for the disease. The PPES were of low quality and our borders are porous making it difficult to screen travelers. (District Director of Health Service 4)

## Discussion

The current study addresses an important global public health topic, EVD surveillance and response preparedness in the context of health system strengthening. The main findings from this study revealed that there are still major gaps in terms of proper functioning of disease surveillance and response preparedness after a decade of IDSR implementation in Ghana. These problems range from validity and quality of suspected EVD data; to problems regarding specimen taking, analysis, and confirmation and feedback; to problems of inadequate resources. Though some countries in SSA have shown some improvements in disease surveillance activities with increased potential for rapid dissemination of information (11, 14, 16, 31), findings from the current study in northern Ghana rather revealed that late case detection and delays to provide health care services to suspected Ebola patients at health facilities are common practices leading to preventable deaths.

There have been reported cases of clinically diagnosed EVD in northern Ghana. It is reassuring that none of the 10 suspected cases in UER was confirmed. However, the eight deaths among the suspected cases due to other causes are a real problem. Largely, it revealed challenges regarding reliability of clinically diagnosed data, accuracy, staff competencies, and commitment. The reported challenges with the rumoured EVD cases are likely attributable to negative attitudes of staff, lack of commitment, fear and panic, lack of PPE, and general lack of skills and competency among health workers, as previously reported in SSA countries (11, 13).

Previous studies in northern Ghana indicated that IDSR strategy has contributed to increased surveillance report submission as well as analysis of surveillance data (20). This is similar to the current findings, which indicated that surveillance data has been reported regularly using the IDSR weekly forms. Specifically for Ebola surveillance, data reporting is through mobile phones. This is an important approach adopted in the health system to improve reporting timeliness and response.

### EVD surveillance core functions and response preparedness

The findings revealed problems in EVD case screening and detection in northern Ghana. The factors contributing to the difficulties in screening and case detection are lack of appropriate equipment (e.g. infra-red thermometers) and problems of overlapping signs and symptoms which mimic other diseases such as malaria, which is the most prevalent disease in Africa. This revelation supports similar reports from SSA countries (14, 16, 20, 31). Except for consulting room registers for capturing patient's personal and clinically diagnosed information, there are no specific registers for recording Ebola cases. The non-existence of such a register contributes to misreporting of surveillance data. The study also revealed difficulties in the confirmation of clinically diagnosed cases due to ill-equipped laboratories and overall limited laboratory capacity. Appropriate implementation of the IDSR strategy components remains a challenge to be resolved. Thus, the possibility of missing the opportunity for early detection and outbreaks due to lack of skills, competencies, and general fear among health workers must be considered a real public health problem. This has been confirmed in the recent outbreak of Ebola in West Africa, where many of the cases were not identified or confirmed on time (32). This is similar to the findings in our study in which none of the suspected EVD cases were confirmed. As a wake-up call, better public information and communication on Ebola, improvement in staff negative attitudes and skills, competencies, and commitment are needed urgently for well-functioning surveillance and response preparedness.

It was repeatedly reported by the informants that weekly IDSR forms were available and accessible, which is likely to contribute to report submission but does not guarantee the quality of data on specific diseases. Besides, EVD data is reported mainly through mobile phone call first before completion of IDSR forms with potential to affect data verification and validation. To address this problem, district and regional level health workers mostly visit the disease control office to cross-check the electronic data compared with the paper-based IDSR data. In previous studies, IDSR forms were rather unavailable at the health facility level (20, 33). Considering the high case fatality rate of EVD, the reported initiative of using mobile phone calls or texting to alert the next level of the health system should be deemed innovative and encouraging. In addition, the electronic reporting on disease surveillance data is another strategy that ensures that data in readily usable form is available. This would also reduce the problems of lack of transport, fuel, and traveling from health facilities to districts and from districts to the region for reports submission. Quality data can improve analysis capability, data availability for comparison, early warning based on increasing trends, and response preparedness. This has been reported in previous studies from developing countries that electronic reporting of surveillance data has a potential to improve early detection of emerging and re-emerging diseases (13, 20, 31, 34).

The district hospital remains the first referral point of contact with the population for suspected EVD cases. However, feedback to periphery facilities is still rare and irregular in most of SSA except during epidemics (14, 16, 20, 31). Disease surveillance feedback has been described as one of the most important activities for improving health workers capacities and performance (35). This study has revealed that health workers at the district level receive electronic feedback through email system while community durbars or gatherings are platforms for surveillance information dissemination to the population. The health facilities (e.g. hospitals, health centres, and clinics) did not have access to feedback immediately except through their district directors.

### EVD surveillance support functions and response preparedness

The findings from this study demonstrate that supervision for surveillance at the district or hospital is rather irregular and also not for Ebola, which supports previous findings (13, 14). Although there is some improvement in the training and employment of disease control officers, they remain inefficient and ineffective. The informants confirmed the existence of problems such as inadequate staff, inadequate funding, frequent staff turnover, low-quality PPEs, limited resources, and poorly equipped laboratories seriously affecting disease surveillance activities. Similar findings have been reported in previous studies in Ghana (20, 36).

### EVD surveillance and response preparedness satisfaction

The findings from this study confirm that surveillance activities for EVD are not satisfactory. Though there is good will from the government, international community, and the national health authorities to provide resources, the PPEs are generally of low quality. The informants cited the porous nature of the country borders, ineffective screening, fear and panic among health workers, and lack of laboratory capacity as reasons for their conclusion. Beside, health workers are helpless due to their overreliance on God for Ebola prevention and strong cultural practices (e.g. burial processes).

The study has some limitations. First, as the investigations were limited to only UER, findings are not representative of the entire health system in Ghana. Second, most of the information was self-reported among health workers and is thus likely influenced by perspective of being health workers. In addition, there was no true Ebola case in the study to determine the real response preparedness.

## Conclusion

In conclusion, although the IDSR system has improved availability of surveillance reporting, EVD surveillance is still insufficient, particularly the lack of registers for Ebola data, inadequate PPEs, burial practices, lack of laboratory capacity, and delays in specimen taking. The Ebola epidemic is a wake-up call for early case detection and response preparedness to prevent outbreaks and spread. This topic remains a neglected public health issue in SSA, including Ghana. Increasing attention and reaching out to other stakeholders is essential for the overall functioning of the health system. Thus, more efforts on disease surveillance and prevention activities are urgently needed.
